# Spatial Patterns of Aflatoxin Levels in Relation to Ear-Feeding Insect Damage in Pre-Harvest Corn

**DOI:** 10.3390/toxins3070920

**Published:** 2011-07-21

**Authors:** Xinzhi Ni, Jeffrey P. Wilson, G. David Buntin, Baozhu Guo, Matthew D. Krakowsky, R. Dewey Lee, Ted E. Cottrell, Brian T. Scully, Alisa Huffaker, Eric A. Schmelz

**Affiliations:** 1 USDA-ARS, Crop Genetics and Breeding Research Unit, Tifton, GA 31793, USA; Email: jeff.wilson@ars.usda.gov; 2 Department of Entomology, University of Georgia, Griffin, GA 30223, USA; Email: gbuntin@uga.edu; 3 USDA-ARS, Crop Protection and Management Research Unit, Tifton, GA 31793, USA; Email: baozhu.guo@ars.usda.gov (G.D.B.); brian.scully@ars.usda.gov (B.T.S.); 4 USDA-ARS, Plant Science Research Unit, Raleigh, NC 27695, USA; Email: matt.krakowsky@ars.usda.gov; 5 Department of Plant and Soil Sciences, University of Georgia, Tifton, GA 31793, USA; Email: deweylee@uga.edu; 6 USDA-ARS, Southeast Fruit and Tree Nut Research Laboratory, Byron, GA 31008, USA; Email: ted.cottrell@ars.usda.gov; 7 USDA-ARS, Center for Medical, Agricultural, and Veterinary Entomology, Gainesville, FL 32608, USA; Email: Alisa.Huffaker@ars.usda.gov (A.H.); Eric.schmelz@ars.usda.gov (E.A.S.)

**Keywords:** edge effect, maize weevil, stink bug, corn earworm, aflatoxin, insect damage, aflatoxin correlation

## Abstract

Key impediments to increased corn yield and quality in the southeastern US coastal plain region are damage by ear-feeding insects and aflatoxin contamination caused by infection of *Aspergillus flavus*. Key ear-feeding insects are corn earworm, *Helicoverpa zea*, fall armyworm, *Spodoptera frugiperda*, maize weevil, *Sitophilus zeamais*, and brown stink bug, *Euschistus servus*. In 2006 and 2007, aflatoxin contamination and insect damage were sampled before harvest in three 0.4-hectare corn fields using a grid sampling method. The feeding damage by each of ear/kernel-feeding insects (*i.e*., corn earworm/fall armyworm damage on the silk/cob, and discoloration of corn kernels by stink bugs), and maize weevil population were assessed at each grid point with five ears. The spatial distribution pattern of aflatoxin contamination was also assessed using the corn samples collected at each sampling point. Aflatoxin level was correlated to the number of maize weevils and stink bug-discolored kernels, but not closely correlated to either husk coverage or corn earworm damage. Contour maps of the maize weevil populations, stink bug-damaged kernels, and aflatoxin levels exhibited an aggregated distribution pattern with a strong edge effect on all three parameters. The separation of silk- and cob-feeding insects from kernel-feeding insects, as well as chewing (*i.e.*, the corn earworm and maize weevil) and piercing-sucking insects (*i.e.*, the stink bugs) and their damage in relation to aflatoxin accumulation is economically important. Both theoretic and applied ramifications of this study were discussed by proposing a hypothesis on the underlying mechanisms of the aggregated distribution patterns and strong edge effect of insect damage and aflatoxin contamination, and by discussing possible management tactics for aflatoxin reduction by proper management of kernel-feeding insects. Future directions on basic and applied research related to aflatoxin contamination are also discussed.

## 1. Introduction

Aflatoxin contamination in post harvest corn presents a serious health problem for human food, animal feed, and ethanol feedstocks from warm temperate, subtropical, and tropical regions worldwide [1,2]. Aflatoxins are produced by filamentous *Aspergillus flavus* Link ex Fries, and *A. parasiticus*, which threaten certain human food and animal feed sources grown under the warm environmental conditions [3,4,5]. Reduction of aflatoxin contamination is a long-term goal for corn, peanut, and other crops in these regions. Reducing biotic and abiotic stresses and breeding for insect and aflatoxin resistance has been part of the integrated approaches taken to reduce aflatoxin contaminations in corn production [6]. The roles of Lepidopteran pests [including corn earworm, *Helicoverpa zea* (Boddie) (Lepidoptera: Noctuidae), and fall armyworm, *Spodoptera frugiperda* (JE Smith) (Lepidoptera: Noctuidae)] in aflatoxin contamination in corn have been documented in recent decades [7,8,9,10]. However, the role of kernel damage by maize weevil [*Sitophilus zeamais* Motschulsky (Coleoptera: Curculionidae)] and stink bugs [*i.e*., the brown stink bug, *Euschistus servus* (Say), the southern green stink bug, *Nezara viridula* (L.), and the green stink bug, *Chinavia *(*Acrosternum*) *hilare* (Say) (Heteroptera: Pentatomidae)] in aflatoxin accumulation are still not clear. In addition, details in spatial distribution of insect pests, associated kernel damage, and aflatoxin contamination in a corn field are still not well understood. Recent efforts by our entomological group in 2005 demonstrated that spatial patterns of maize weevil infestation in a pre-harvest corn field were correlated to aflatoxin levels, but not correlated to stink bug damage throughout the corn field at pre-harvest [6]. 

**Figure 1:**
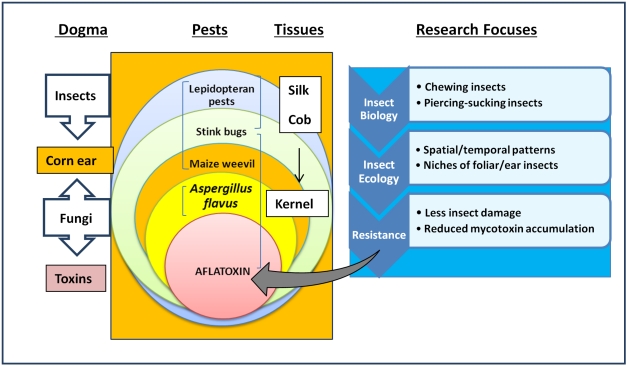
Ecological interactions among insect pests and aflatoxin contamination in corn ears between flowering and harvest in the Southeastern Coastal Plain Region of the U.S.

We have taken a comprehensive approach in recent years to understand ecological details related to aflatoxin contamination by separating cob- and silk-feeding insect damage from kernel-feeding insects and their damage at the preharvest corn fields (Figure 1). The current study is one in a series to determine spatial patterns of ear- and kernel-feeding insect damage and their contributions to aflatoxin accumulations in corn ears. Objectives of the study were to: (1) determine the spatial distribution patterns of insect populations and their damage; and (2) examine the correlation between insect population and damage to aflatoxin accumulation in the corn fields.

## 2. Results and Discussion

### 2.1. Results

#### 2.1.1. Grid-Sampling Data of 2006 Data

The five response variables (*i.e*., corn husk coverage, corn ear worm damage, number of maize weevils, and stink bug-discolored kernels) varied throughout the corn field at pre-harvest. Corn husk coverage varied significantly among the rows (*F* = 4.16, *df* = 4, 355, *P* = 0.003), but not among the sampling points within rows (*F* = 1.38, *df* = 16, 355, *P* = 0.15). Corn earworm damage differed among rows (*F* = 3.26, *df* = 4, 355, *P* = 0.01), and also among the sampling points within rows (*F* = 1.9, *df* = 16, 355, *P* = 0.02). Maize weevil infestation differed among the rows (*F* = 9.61, *df* = 4, 355, *P* = 0.0001), and also among the sampling points within rows (*F* = 2.69, *df* = 16, 355, *P* = 0.0005). Stink bug damage did not differ among the rows (*F* = 1.89, *df* = 4, 355, *P* = 0.11), but differed among the sampling points within rows (*F* = 3.48, *df* = 16, 355, *P* = 0.0001). Because of the variation in aflatoxin levels among samples collected in 2006, aflatoxin levels from each sampling point was not significantly different either among rows (*F* = 1.48, *df* = 4, 55, *P* = 0.22), nor among the sampling points within rows (*F* = 0.81, *df* = 16, 55, *P* = 0.67).

**Figure 2:**
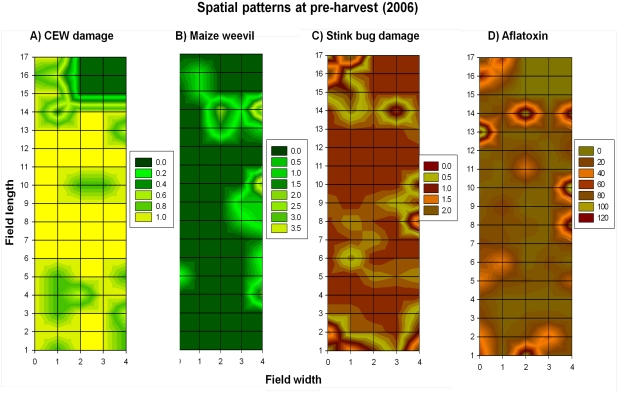
The spatial distribution patterns (*i.e.*, aggregated distribution and edge effect) of corn earworm (CEW) damage (**A**), the number of maize weevils (**B**), percentage of stink bug-damaged kernels (**C**), and aflatoxin levels (ppb) (**D**) in the corn field at pre-harvest in 2006. Aflatoxin levels (ppb) (**D**) were not to corn earworm damage (**A**) (*r* = 0.1, *P* = 0.37, *n* = 76), but positively correlated to the number of maize weevils (**B**) (*r* = 0.25, *P* = 0.03, *n* = 76), and the percentage of stink bug-damaged kernels (**C**) (*r* = 0.36, *P* = 0.001, *n* = 76).

Correlations among the five indices in 2006 are summarized in Table 1. Aflatoxin level was not correlated to either husk coverage or corn earworm damage, but was positively correlated to both stink bug damage, and the number of maize weevils (Table 1). The contour maps of the 2006 grid-sampling data (*n* = 76) at pre-harvest showed that maize weevil infestation and stink bug damage, and aflatoxin levels were aggregated throughout the corn field, and a strong edge effect (Figure 2A–D). Lepidopteran insect (e.g., corn earworm and fall armyworm) caused cob damage was not correlated (*r* = 0.1, *P* = 0.37; Table 1) to aflatoxin levels in corn samples (Figure 2A), which is different from previous contamination [7,8,9,10]. As indicated in Table 1, the number of maize weevils (Figure 2B) and percentage of discolored kernels (Figure 2C) were correlated to aflatoxin levels (Figure 2D). Because aflatoxin level was not correlated to husk coverage as shown in Table 1, the contour graph of husk coverage is not presented here. Please refer to Table S1 for the complete original dataset of 2006.

**Table 1:** Correlation between aflatoxin levels and husk coverage, insect infestation (maize weevil) and insect damage (corn earworm and stink bug damage) in 2006 (*n* = 76)*.

	Husk Coverage	Discolored Kernels	Maize Weevil	Aflatoxin (ppb)
Corn earworm	−0.09	−0.17	0.08	0.1
	0.46	0.15	0.49	0.37
Husk coverage		0.12	−0.01	−0.12
		0.31	0.93	0.29
Stink bug-			0.05	0.36
discolored kernels			0.68	0.001
Maize weevil				0.25
				0.03

* In each table cell, top value is the Pearson’s correlation coefficient (*r*) value, and bottom value is *P* value.

#### 2.1.2. Grid-Sampling Data of 2007

The five parameters assessed varied throughout all three corn fields. Corn earworm damage varied significantly among rows (*F* = 4.13, *df* = 4, 300, *P* = 0.003), and among fields (*F* = 18.65, *df* = 2, 1271, *P* = 0.0001), but not among sampling points within rows (*F* = 1.37, *df* = 16, 300, *P* = 0.16). Maize weevil infestation varied significantly among rows (*F* = 16.36, *df* = 6, 1271, *P* = 0.0001), among sampling points within rows (*F* = 1.88, *df* = 16, 1271, *P* = 0.02), and among fields (*F* = 16.87, *df* = 2, 1271, *P* = 0.0001). Percentage of stink bug-damaged kernels varied significantly among rows (*F* = 57.7, *df* = 6, 1271, *P* = 0.0001), among the sampling points within rows (*F* = 28.44, *df* = 16, 1271, *P* = 0.02), and among fields (*F* = 30.19, *df* = 2, 1271, *P* = 0.0001). Husk coverage varied significantly among rows (*F* = 2.43, *df* = 6, 1271, *P* = 0.02), among sampling points within rows (*F* = 2.06, *df* = 16, 1271, *P* = 0.008), and among fields (*F* = 14.12, *df* = 2, 1271, *P* = 0.0001). Aflatoxin levels in the ground corn samples did not vary significantly among the three fields (*F* = 2.39, *df* = 2, 238, *P* = 0.09), nor by rows, or sampling points within rows (*P* values > 0.05), because of the great variation among the sample data (Figure 5A–C). 

**Figure 3:**
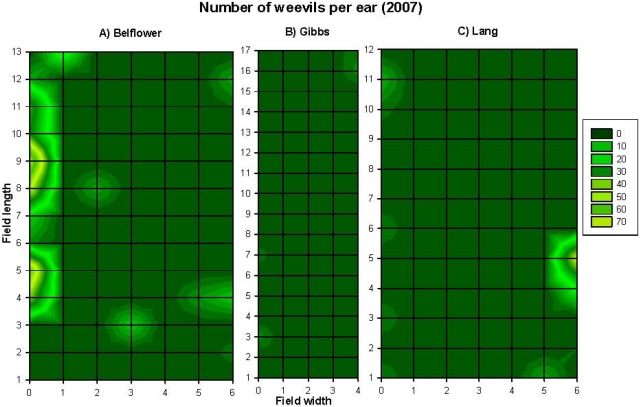
The spatial patterns (*i.e*., aggregated distribution, and edge effect) of the number of maize weevils in the corn fields on the three research farms at preharvest in 2007. (**A**) Belflower Farm, (**B**) Gibbs Farm, and (**C**) Lang Farm. The grid size was the same in all three fields, although the grid size was smaller in the graph.

**Figure 4:**
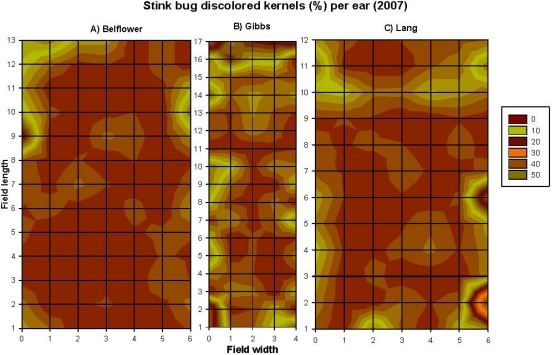
The spatial patterns (*i.e*., aggregated distribution, and edge effect) of the stink bug-discolored kernels (%) in the corn fields on the three research farms at preharvest in 2007. in the corn fields on the three farms in 2007; (**A**) Belflower Farm, (**B**) Gibbs Farm, and (**C**) Lang Farm. The grid size was the same in all three fields, although the grid size was smaller in the graph.

**Figure 5:**
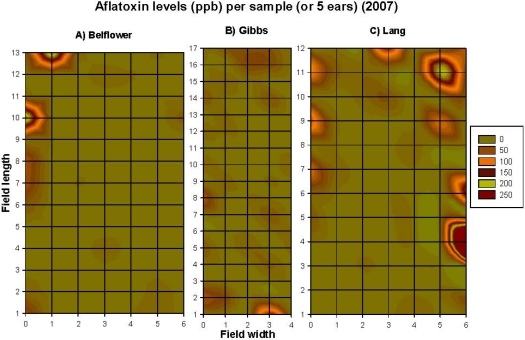
The spatial patterns (*i.e*., aggregated distribution, and edge effect) of the aflatoxin contamination (ppb) in the corn samples on the three research farms at preharvest in 2007. (**A**) Belflower Farm, (**B**) Gibbs Farm, and (**C**) Lang Farm. The grid size was the same in all three fields, although the grid size was smaller in the graph.

Correlations among the recorded parameters (combined data from the three fields, *n* = 260) in 2007 are summarized in Table 2. Aflatoxin level was positively correlated to corn earworm damage, maize weevil infestation level, and percentage of stink bug-damaged kernels (Table 2). However, when the correlation was assessed within each of the three fields, the results varied among the three fields. The contour map showed that the number of maize weevils (Figure 3A–C), percentage of discolored-kernels (Figure 4A–C), and the levels of aflatoxin contamination (Figure 5A–C) had a strong edge effect in all three corn fields examined. In 2007, because husk coverage was not correlated to aflatoxin levels, and corn earworm damage had a very low correlation (*r* = 0.14) with aflatoxin with the combined dataset (Table 2), the data of these two parameters were not presented here, however, all original data are provided as Table S1 in Supporting Information section. Corn earworm damage was only positively correlated to aflatoxin level on the Belflower Farm: *r* = 0.22, *P* = 0.03, *n* = 91, but not on the other two farms (Gibbs Farm: *r* = 0.05, *P* = 0.62, *n* = 85; Lang Farm: *r* = 0.14, *P* = 0.22, *n* = 84).

**Table 2:** Correlation between aflatoxin levels and husk coverage, insect infestation (maize weevil) and insect damage (corn earworm and stink bug) in 2007 (*n* = 260)*.

	Husk Coverage	Discolored Kernels	Maize Weevil	Aflatoxin (ppb)
Corn earworm	−0.13	0.16	0.28	0.14
	0.04	0.009	0.0001	0.02
Husk coverage		−0.08	−0.11	0.01
		0.2	0.09	0.84
Stink bug-			0.14	0.18
Discolored kernels			0.02	0.003
Maize weevil				0.19
				0.002

* In each table cell, top value is the Pearson’s correlation coefficient (*r*) value, and bottom value is *P* value.

However, when assessed within each of the fields, the results varied. The Belflower Farm had the largest maize weevil infestation (Figure 3A), while the Gibbs Farm (Figure 3B) had the smallest. The maize weevil and aflatoxin level was correlated among two of the three farms [Belflower Farm: *r* = 0.44, *P* = 0.0001, *n* = 91 (Figure 3A and Figure 5A); Gibbs Farm: *r* = −0.04, *P* = 0.7, *n* = 85 (Figure 3B and Figure 5B); and Lang Farm: *r* = 0.30, *P* = 0.006, *n* = 84 (Figure 3C and Figure 5C)]. Similarly, aflatoxin level was highly correlated to stink bug-damaged kernels on all three farms [Belflower Farm: *r* = 0.46, *P* = 0.0001, *n* = 91 (Figure 4A and Figure 5A); Gibbs Farm: *r* = 0.35, *P* = 0.001, *n* = 85 (Figure 4B and Figure 5B); and Lang Farm: *r* = 0.26, *P* = 0.02, *n* = 84 (Figure 4C and Figure 5C)]. Please refer to Table S1 for the complete original dataset of 2007.

### 2.2. Discussion

A number of studies have extensively examined the timing and inoculation methods [11,12], and the genetics of aflatoxin resistance in corn germplasm [13,14,15,16,17]. Unfortunately, the field ecology of insect infestations and their damage, as well as the ecological information on toxigenic fungus infection is still fragmentary. Several studies have described the putative role of insects in aflatoxin contamination [4,7,8,18,19]; these studies demonstrate the complexity of the problem and the importance of understanding ecological interactions for mitigating infestations. The current study is one of the first multiple-year studies utilizing high resolution grid-sampling technique throughout a field to assess the spatial patterns of maize weevil infestations and other ear-feeding insect damage in relation to aflatoxin contamination. Data presented here showed that patterns of maize weevil infestation, percentage of stink bug-damaged kernels, and aflatoxin levels in the grain were distributed in aggregations throughout a field with a strong edge effect at pre-harvest. The results from the present study confirmed that the previous observations by [9,10] that maize weevil can play an important role in aflatoxin contaminations in corn production. The findings also confirmed results from the previous report from our group [6] that maize weevil populations and aflatoxin contamination had a strong edge effect and aggregated distribution patterns in 2005.

The separation of tissue-specific insect feeding (*i.e*., silk and cob feeding versus kernel feeding) in relation to their contributions to aflatoxin contamination indicated that the kernel-feeding insect damage might be critical, although cumulative insect damage is likely to influence aflatoxin accumulations. Maize weevil population and stink bug damage were positively correlated to aflatoxin levels in both years, while silk- and cob-feeding damage by the corn earworm and the fall armyworm was positively correlated to aflatoxin level only in the combined data of 2007, thereby suggesting a weaker association. The findings suggest kernel-feeding damage at pre-harvest might be more important than the silk- and cob-feeding damage by the corn earworm and fall armyworm post flowering. The findings provided more details in addition to Widstrom *et al*. [7,18] who reported that the ear-feeding lepidopteran insects are one of the important factors for aflatoxin contamination in corn fields at pre-harvest under warm climatic conditions. 

In addition to insect damage, the variation between 2006 and 2007, as well as among the fields in 2007 confirmed the elusive nature of aflatoxin contamination problem in crop production under warm climate conditions. The variation in aflatoxin levels among sampling sites could be the results of abiotic factors (e.g., temperature, rainfall, and net evaporation rate), in addition to the effect of biotic stress factors (e.g., various insect feeding damage, and disease infections). These factors should be further examined in future studies.

## 3. Experimental Section

### 3.1. Fields

Three approximately 0.4 hectare (one acre) fields were planted with a commercial soft dent corn hybrid ‘DeKalb DK64-10’ on three University of Georgia and USDA-ARS research farms located at Tifton, GA to assess the patterns of ear-feeding insect infestations, associated insect damage, and aflatoxin contamination at pre-harvest. The field on the Gibbs Farm was planted on April 17, 2006 for the first year, and the three fields on the Belflower, Gibbs and Lang Farms were planted on March 25, 2007 for the second year. All corn fields were maintained with the conventional agronomic practices for fertilization and herbicide applications, but no insecticides were applied during the growth seasons. 

### 3.2. Corn Sampling

When corn kernels had reached optimal moisture content for storage (approximately 15% moisture), a 9 × 9-m (or 8 × 8-m depending on field dimensions) grid was overlaid across the entire field. Fields were sampled at the same maturity (approximately 18–20 weeks after planting) in September 2006 and 2007. At the pre-harvest sampling dates (05 September for Gibbs Farm in 2006; and 10 September for Gibbs Farm, 11 September for Lang Farm, and 12 September for Belflower Farm in 2007), five ears were hand harvested from each grid point. Insect damage and corn ear phenotypical features were assessed and then ears were shelled and ground for aflatoxin analysis. There were 76 sampling points (17 points × 2 rows + 14 points × 3 rows) in the field on the Gibbs Farm in 2006, because of the irregular shape of the field. In 2007, the three fields on the Belflower, Gibbs, and Lang Farms had 91 (7 × 13), 85 (5 × 17), and 84 (7 × 12) sampling points, respectively. 

### 3.3. Insect and Associated Damage Sampling

Pre-harvest ear damage caused by natural infestations of three insect pests was assessed. First, husk coverage was assessed by assigning a binomial rating of open husk = 0 or closed husk = 1. Next, ear damage by corn earworm/fall armyworm complex was rated using a similar binomial damage rating scale (*i.e*., 0 = no damage; and 1 = damaged corn cob) as a single index of lepidopteran insect damage. Because maize weevils move into the corn field when kernel moisture is below 22% [20], the damage assessment is not reliable at the sampling time when the kernel moisture was at 15%. Maize weevil populations were determined by enumerating the number of adult maize weevils per ear. For assessing stink bug damage, the number of discolored kernels per ear was enumerated. To calculate percentage of the discolored kernels, the total number of kernels per ear was estimated using a single representative ear from each sampling point.

### 3.4. Aflatoxin Quantification

The five ears harvested from each sampling point were dried for at least 7 d at 61 °C and then shelled and pooled by sampling location, and finally ground using a Romer Series II^®^ mill (Romer Laboratories, Inc., Union, MO). The concentration of aflatoxin in ground grain was determined by the VICAM Aflatest system (Watertown, MA) using the fluorometer method. This procedure can detect aflatoxin contamination as low as 1 ng/g. Briefly, a 100-g sample of the ground corn was used in this analysis for aflatoxin analysis. The quantification of aflatoxin level in a sample was repeated with a smaller sample (10 g sample) if a high level (>300 ppb) of aflatoxin was detected in a sample. The reason for using a smaller sample size for the repeat was to ensure the aflatoxin readings were within the linear part of the standard curve. When assays were repeated, the second measurement (with a multiplication factor of 10) was used. 

### 3.5. Experimental Design and Data Analysis

Treatments were arranged in a completely randomized design. The three 0.4-hectare corn fields on the three research farms were modeled as three replications of the experiment. Spatial patterns of the insect damage and aflatoxin levels were compared using ANOVA (PROC MIXED, SAS Institute, Cary, NC). Correlations between aflatoxin levels and three types of insect data were determined using Pearson Correlation Coefficients (PROC CORR, SAS Institute, Cary, NC). The data were plotted using SigmaPlot (SystatSoftware Inc., Richmond, CA) to illustrate dispersion of response variables. 

## 4. Conclusions and Further Research Directions

Although significant progress in understanding the aflatoxin problem has been made [21], aflatoxin contamination of corn and other crops is still a serious agricultural problem [6,22]. Among all aspects of the research related to aflatoxin reduction, host plant resistance to aflatoxin accumulation and breeding efforts are making significant progress, whereas quantifying the ecological interactions of both biotic and abiotic factors has been lagging behind. The present study indicated that insect herbivory affected the pre-harvest aflatoxin contamination in the Southeastern Coastal Plain of the US. Information on the contributions of the spatial versus temporal field patterns of ear- and kernel-feeding insect damage and aflatoxin accumulations is a first step toward identifying the key factors within the ecological complex in corn cropping systems. This spatial correlation study is an initial documentation of the differential impact of chewing and piercing/sucking insect damage on kernels and cobs in relation to aflatoxin contamination.

The current study shows a highly aggregated pattern of cob- and kernel-feeding insect damage, and aflatoxin contamination, as well as a strong edge effect of both insect damage and aflatoxin levels. Aflatoxin levels were better correlated to the kernel-feeding maize weevils and kernel damage by stink bugs than to cob-feeding corn earworm damage. We hypothesize that the strong edge effect could be caused by an increased susceptibility of plants located on the perimeter compared to those located on the interior. An increased susceptibility corn plants located at the field edge is likely the result of both biotic and abiotic environmental stresses, including high insect (e.g., stink bugs and maize weevil) pressures, and possibly less irrigation throughout a growing season. The combination of abiotic factors and the resulting physiological state of corn plants after pollination in relation to biotic factors (e.g., insect damage) might be the critical in *A. flavus* infection, aflatoxin accumulation, and other ear rot infections. At present, this hypothesis is being examined by our multidisciplinary team, by initially examining the ecological, physiological and biochemical processes that occur during the early stages of ear infection which influence resulting mycotoxin contamination. By examining this system at multiple levels of analyses (including environmental, ecological, physiological, and biochemical) the interactions between maturing corn kernels, insects and mycotoxigenic fungi in the southeastern region of the U.S. will eventually be understood thus enabling strategic improvements. 

In addition to theoretic contribution by forming a new hypothesis, the findings presented here also have practical ramifications for aflatoxin reduction for corn growers in our region. Although it has not been empirically tested, these data suggest that researchers need to consider testing practical prevention and/or management tactics for reducing both insect damage and aflatoxin contamination in further studies. For example: (1) selective application of insecticides or fungicides to field perimeters, which may reduce both insect damage and aflatoxin contamination at the most susceptible field edges; (2) separate harvest of the perimeter and interior portions of a corn field thereby segregating grain by potential for aflatoxin infestation; and (3) selecting larger fields that are relatively square in shape to decrease the perimeter to area ratio of the field. 

## Supplementary Files

Supplementary File 1: Table S1 (XLSX, 85 KB)
